# The hidden dimension of open science: “Peopleware”

**DOI:** 10.1016/j.patter.2021.100385

**Published:** 2021-11-12

**Authors:** Claudia Bauzer Medeiros

**Affiliations:** 1 Institute of Computing, UNICAMP. Av Albert Einstein 1251, Campinas, SP 13081-852, Brazil

## Abstract

In discussing open science, one forgets that its key concept is *collaboration*, which may be either accelerated or hampered by digital technologies. Collaboration in personal interactions is hard; how much harder, then, is it to collaborate across temporal, geographical, or cultural barriers? Open science can be seen as a worldwide case study on peopleware—a major source of costs, but a huge asset.

## Main text

### Introduction

The UNESCO draft recommendation on open science,^1^ which took 3 years to prepare, with inputs from member countries and scientific societies, is the most visible worldwide political effort toward the open science movement. Though researchers recognize the value of collaboration as a means to improve or accelerate knowledge discovery, all acknowledge that there are challenges and barriers to achieve cooperation. Such challenges vary from the individual to institutions and regions, involving economic, technological, or sociocultural factors. The lack of a consensual definition also hampers implementation and compliance.

Some point out that, since open science is an enabler of “collaboration without barriers,” it actually dates back to the 17^th^ century, having been born with the first journals and records of correspondence among scientists. This notwithstanding, the more widespread understanding is that it is associated with *digitally enabled scientific collaboration*, through sharing the *digital* objects associated with research.

This stress on the digital points out our dependence on technological enablers and the very many challenges posed by the many kinds of digital divides—social, cultural, economic, educational. This dependence has also prompted the continuous appearance of new kinds of computing infrastructures, standards, and frameworks to support open science. The associated terms “accessible,” “reproducible,” and “reusable” originated research in many fields in computer science and are key to understand this movement and the challenges it poses to information technology. “Reuse” in particular is fraught with undercurrents, since it also implies repurposing, and the fear this will produce undesirable side effects to one’s research. This is an example of the dual ways—advantage, barrier—people react to open science.

### The open science ecosystem

**Figure 1:**
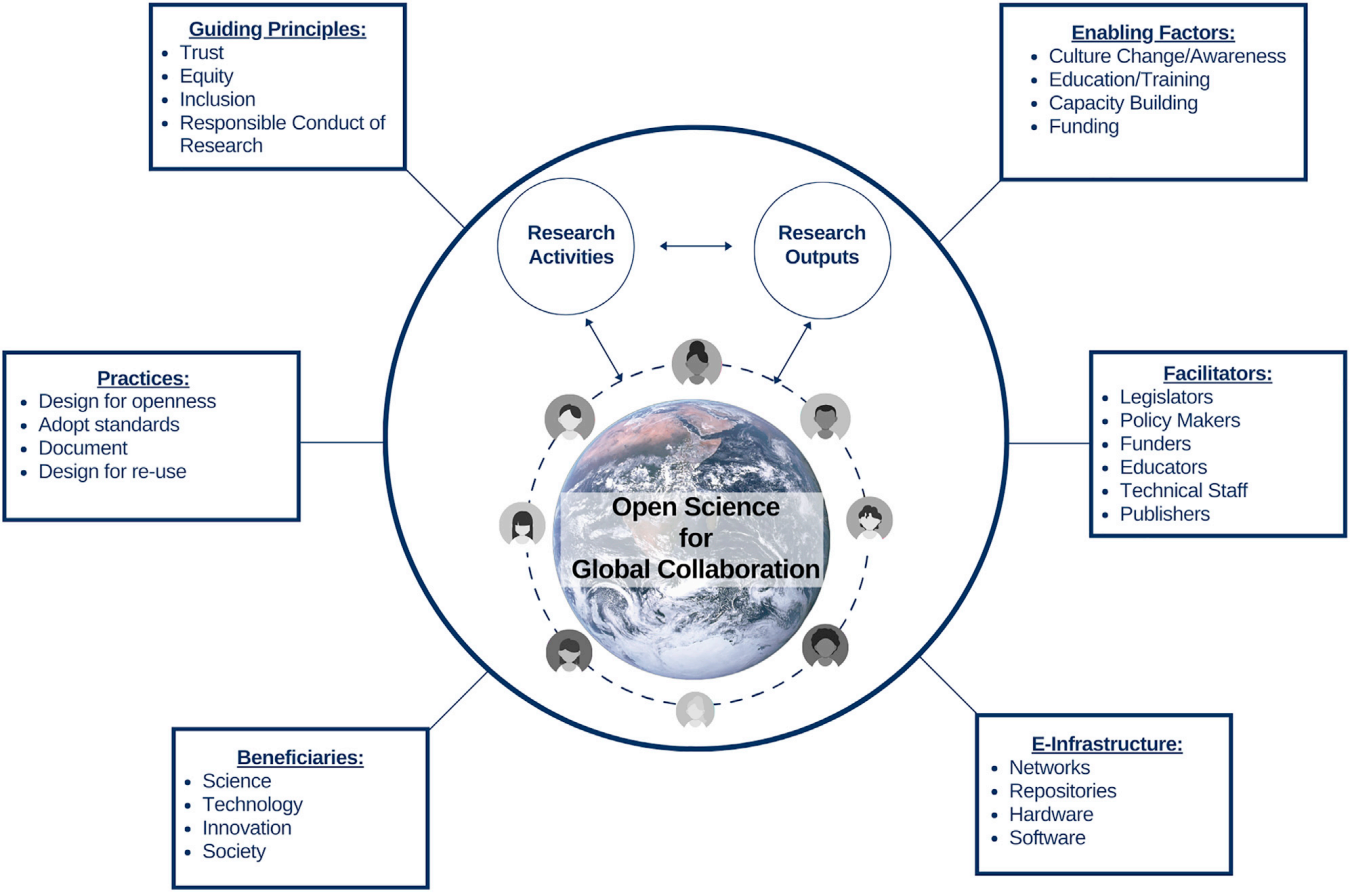
Open science ecosystem Researchers interact globally through sharing and reuse of research activities and outputs. Reproduced from the IAP Report.^2^

The “open science ecosystem” of the InterAcademy Partnership Report,^2^ summarized in Figure 1, places global collaboration at its core. It depicts a virtuous cycle in which researchers share their work (separating the outputs themselves from the activities that lead to them) to enable cooperation through reuse, centered in a continuous exchange of research outputs and practices.

In this ecosystem, we recognize the most commonly cited pillars of open science, which can be summed up as hardware, software, and peopleware. The explicit digital component is restricted to the “E-infrastructure” component, involving hardware, software, networks, and repositories. Peopleware appears in all other components—e.g., as adopters of practices (such as the “design for openness”^3^), or facilitators, or as supported by enabling factors (with education, capacity building, adequate funding, and culture change).

A central focus of this ecosystem is the sharing of the outputs and the outputs themselves. One of these outputs—open publications—is often considered synonymous to open science and is at the center of an ongoing debate among scientists, publishers, and funders to establish new economic models for publishing “for free.” PlanS, for one, is an example of such a model, with its defenders and detractors. Open computational processes and open software, and the need for making them FAIR, represent a second important dimension of the ecosystem, as discussed in, for instance, the work of Katz et al.^4^

While the openness of publications and software (and the associated costs) are understood by researchers, data sharing—and open data in particular—are concepts whose realization are still in their infancy, with implementation, cultural, and political barriers. Perhaps one reason is that there are consensual understandings of what a publication is and how to share it. By the same token, the notion of software sharing through repositories is generically grasped by all who produce or consume research software. Incongruously, though everyone uses and produces data continuously, there are widely varying understandings of the concept of data. Consequently, data sharing is perhaps the biggest digital barrier in the open science ecosystem, not only because data is an asset and a commodity but also because many scientists are unsure of what kind of data they may share. While scientists in some domains (e.g., -omics) have a long tradition of data sharing, others (e.g., engineering) are unsure about how to proceed. This varies considerably across disciplines and geographic regions.

### Case study: The creation of the São Paulo open research data network

In 2017, recognizing the impact of open data on research, FAPESP (the São Paulo Research Foundation) launched a multi-year initiative to implement open data policies. FAPESP is a pioneer in open science actions ^5^. Its open data plan was implemented along two main axes: (1) requiring compulsory data management plans (DMPs) upon proposal submission, effective November 2017, and (2) sponsoring a working group (WG) to create a statewide network of institutional open research data repositories.

#### Compulsory DMPs and culture change

In its first stage, DMPs were compulsory only upon submission of large multi-year collaborative projects; by September 2020, they were extended to practically all of FAPESP funding lines. In 2017, virtually no one had heard about DMPs in Brazil or understood their role in a project. Considering that FAPESP receives annually on average 22,000–25,000 grant proposals, in all domains, this brought about a large—and sudden—culture change among researchers in the State of São Paulo. Since these plans are evaluated upon grant submission, and afterward their compliance (and evolution) is also analyzed within periodical reports, they soon became important elements in the quest for funding. Initially considered another bureaucratic demand, to be written and then forgotten, researchers are coming to realize their usefulness. This, in turn, is generating demands from researchers to their institutions—e.g., in assistance to write a plan or to help manage data for sharing. Even more important for culture change than the plans themselves is the message conveyed: in advancing knowledge discovery, open quality data is as important as open quality papers.

#### The São Paulo open research data network

FAPESP recognized that it is useless to ask people to plan data management if institutional repositories are not available. Thus, it instituted a WG with representatives from all seven public universities of the State of São Paulo to design and implement the infrastructure for open data. In 2018, the WG was joined by the Informatics Research Center of the Brazilian Agricultural Research Corporation, EMBRAPA. This network, publicly available since December 2019, hosts scientific data from research produced by the network members in all scientific domains; its design and construction involved more than 100 people across the institutions—administrators, legal officers, IT staff, computer scientists, librarians, and researchers from multiple fields.

The WG, coordinated by myself, started working in mid-2017 and comprised a mix of librarians and computer scientists with experience in interdisciplinary research. The entire enterprise took advantage from the accumulated expertise and experience of members of the Research Data Alliance (RDA),^6^ with whom we interacted throughout the entire period, including for advice on *what not to do*.

**Figure 2:**
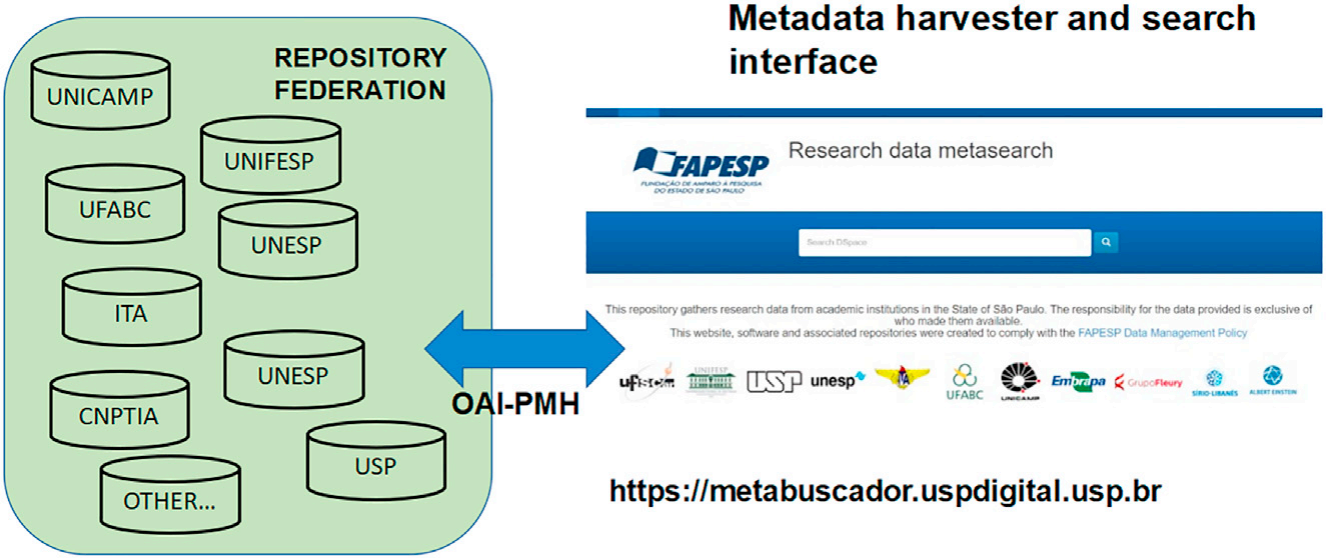
Overview of the repository network A federation of independent member repositories, one per institution, publishes metadata of their files through a common interface. Each institution created a board to oversee its repositories and data management policies (see lessons learnt).

The network was conceived and implemented with openness and extensibility in mind. For this reason, early on we chose a solution of a federation of independent repositories, each of which would publish the metadata of the data files stored therein (see Figure 2). Experts from the University of São Paulo (USP) implemented a metadata harvester that daily collects these metadata from the federation “members” and publishes them through a common interface, shown in Figure 2. Any academic institution in the State of São Paulo can now join the network, provided it complies with exposing a minimum set of metadata fields through the OAI-PMH protocol.^7^

#### The COVID-19 DataSharing/BR repository

When the pandemic struck, FAPESP immediately decided that researchers needed a comprehensive open repository of patient data to support data science initiatives for public health associated with COVID-19. It put together a public-private partnership of hospitals and laboratories that agreed to share their data. Under the technical leadership of USP, and thanks to the extensibility of the network, it was possible, in less than one month, to design, create, and deploy this repository, launched in June 2020,^8^ as a federation member. It contains two kinds of pseudonymized data: demographics (gender, year of birth, and region of residency), clinical and laboratory exams, hospitalization information, and additional medical information, when available. By October 2021, it contains open data from 800,000 people and more than 50 million clinical data records, duly pseudonymized, and it has been accessed by people from 24 countries, with thousands of downloads.

### Revisiting peopleware: Some lessons learnt from coordinating the repository network

The creation of the network had three interesting side effects. First, because of institutional engagement, it raised awareness to the advantages and needs of open science in a country yet not familiar with this movement. Second, this in turn attracted researchers, who are becoming slowly engaged in associated activities. Third, being a pioneer effort in Brazil, it is being used as example to start similar initiatives in other parts of the country.

A “federation of repositories” evokes digitally laden terms such as “networks,” “databases,” and “machines” and worries about the associated costs. These are the tip of the iceberg. What, however, were the lessons learnt concerning the rest of the iceberg—namely, what is required peopleware?

#### Establish a permanent governance structure

Open science initiatives are long term and should not depend on the whims of personnel. Permanence assures researchers, who realize that these initiatives are not temporary and thus are worth the effort required (e.g., in curating and documenting data and software). Sound institutional governance percolates up by example (to regional and national levels).

#### Create institutional steering committees

The network participants created such committees, which were assigned a double task: creating and maintaining the institutional repository and providing assistance and training to researchers. In some institutions, this has percolated down to departments that are now creating their own groups to foster open science practices, e.g., in education or chemistry.

#### Embrace diversity

Successful steering groups should include competent people with diverse backgrounds, in particular (1) someone with IT experience, for data centers, networks, and design and maintenance of repositories; (2) someone in library sciences, with experience in cataloging and archiving practices, ideally connected with expertise in data management; (3) people who practice data-centered research; and (4) someone from central administration.

#### Beware diversity for diversity’s sake

Such groups will only work if guided by common principles and composed of competent people who are willing to dedicate extra hours to promote and support the necessary culture change and influence budget decisions.

#### Approach open science initiatives top-down and bottom-up

No individual effort will succeed if the central administration is not convinced of the worth of open science, thereby promoting changes in institutional policies, infrastructure, and staff allocation. Also, no matter how much administrations are imbued with the spirit of open science, it will never take root without engaging the enthusiasm of researchers and staff.

#### Leverage researcher adoption through funder initiatives

Had FAPESP not demanded compulsory DMPs nor instituted the network, open data would perhaps be less widespread in Brazil. Had researchers, as a consequence, not prodded institutions to provide the necessary infrastructure and staff allocation, there would not exist institutional policies to support open science.

#### Take people divides into account

From the start, engage people who know how to navigate an institution’s internal power and political divides. No matter how technically competent, this kind of cultural change requires more than scientific knowledge.

#### Demand trained staff

Our repository network involves 48 campuses and a large research center. All institutions already had sufficient hardware and IT staff to minimally create repositories and perform installation and integration tests. Each institution decided how to create its repositories according to its overall infrastructure, staff know-how, and legal constraints. The common metadata harvester interface was designed and developed by computing experts at USP and made available within the first 6 months of the WG. Had this kind of expertise not been available from the start, the entire project would have taken much longer. Lack of training of some IT staff led to disparities in local implementations.

#### Foster advocacy of open science as a key factor to collaboration and accelerating discovery

Initiatives will succeed if researchers are convinced of the usefulness to their careers. It is not enough that funders make them compulsory: the ones preparing shareable research outputs must believe that this is not yet another useless activity. This entails a continuous effort to convince researchers to use the repositories and help them to do so.

#### Plan for continuous training and instilling trust

Training of researchers and staff at different levels is needed—to embrace and support open science. People will only change their research/work habits if they trust the institutional environment—e.g., to provide archival facilities, to ensure credit is given where it is due. In our case, this prompted librarians to understand data stewardship. Not having been trained as such, the most common attitude is that “data are made from bits” and “publications are made from bits.” Thus, researcher support should be the same. Awareness of the differences is requiring that the more traditional librarians change their outlook on how to perform their activities.

#### Engage all actors

Given a person can play multiple roles in open science, researchers should involve all actors in research planning and design, thereby making all understand the wonderful contributions to knowledge building a project will bring about.

#### Establish an alliance between computer scientists and librarians

Peopleware is an asset but also hampers the ecosystem. Due to its reliance on digital technologies, but also on organization of information, there is often a dispute between IT and library staff on who should “own” an institution’s open science policies and initiatives. Depending on how people are internally organized and connected in an institution, this may severely hamper the progress of any open science initiative.

#### Recognize people

An obvious lesson, which everyone mentions but is hard to practice, because promotion or tenure structures do not recognize how hard it is to practice “open by design” and “plan for sharing.”

### Conclusions: The open secret of open science

Research requires permanent investment. It stands to reason that any kind of initiative toward collaborative research should also cost money. For some reason, there exist expectations that open science will save money. It does, indirectly, through reuse, learning from others, enabling new discoveries and transparency. Of course, there will always be a need for investment in, e,g, E-infrastructure (and maintaining it). A non-negligible cost fraction is culture change, which takes many years and requires time, patience, and enthusiasm of all involved. As such, the open secret of open science is that peopleware is its major and perennial cost—not only the producers of science but the entire ecosystem. It is also its major asset and, as such, should be treated, nurtured, and recognized.
